# Fabrication, Characterization, and Antifungal Activity of Chitosan–Cyproconazole Nanocomposite for Simultaneous Wheat Stem Rust Control and Growth Enhancement

**DOI:** 10.1049/nbt2/6628425

**Published:** 2026-01-02

**Authors:** Jafar Fathi-Qarachal, Seyed Ali Moosawi-Jorf, Maryam Nikkhah, Mansoor Karimi-Jashni

**Affiliations:** ^1^ Department of Plant Pathology, Faculty of Agriculture, Tarbiat Modares University, Tehran, Iran, modares.ac.ir; ^2^ Department of Plant Protection, Faculty of Agriculture, Shahid Chamran University of Ahvaz, Ahvaz, Iran, scu.ac.ir; ^3^ Department of Nanobiotechnology, Faculty of Biological Sciences, Tarbiat Modares University, Tehran, Iran, modares.ac.ir

**Keywords:** chemical treatment, fungal disease, nano-formulation, *Puccinia graminis* f. sp. *tritici*

## Abstract

The stem rust disease caused by obligate biotrophic fungus *Puccinia graminis* f. sp. *tritici* is a worldwide threat to the global wheat production with frequent epidemics leading to widespread reliance on chemical fungicides such as cyproconazole. To reduce fungicide risks on human health and environmental integrity, chitosan nanoparticles (CNPs) and novel chitosan–cyproconazole nanocomposite (Chi‐Cyp) were synthesized. Dynamic light scattering (DLS) and Fourier transform infrared (FTIR) spectroscopy confirmed the size of 80–90 nm and surface charge and uniformity. To evaluate their efficacy against the disease, various concentrations of CNP and Chi‐Cyp were applied via irrigation, foliar spray, and a combination of both methods. Wheat seedlings were treated 24 h prior to inoculation, as well as at 48‐ and 96‐h post‐inoculation with Pgt urediniospores. Phenotypic assessments conducted 2 weeks post‐inoculation revealed that CNPs (100 μg/mL) and Chi‐Cyp (1 μg/mL), along with the positive control cyproconazole (10 μg/mL), significantly suppressed stem rust infection. Quantitative polymerase chain reaction (qPCR) analysis corroborated these findings, demonstrating a substantial reduction in fungal biomass in treated plants. Additionally, the impact of the nanomaterials on plant growth parameters was examined. Notably, Chi‐Cyp treatment at 50 μg/mL significantly enhanced seedling growth, as evidenced by increased shoot and root lengths, and elevated fresh and dry biomass accumulation. This study highlights the potential of the Chi‐Cyp nanocomposite, which contains a 10‐fold lower concentration of cyproconazole, to effectively control stem rust with comparable efficacy to the fungicide alone. These findings underscore the promise of nanotechnology‐based strategies in sustainable plant disease management.

## 1. Introduction

Wheat stem rust, also known as black stem rust, caused by *Puccinia graminis* f. sp. *tritici* (Pgt), is one of the most destructive wheat diseases worldwide [1]. Since stem rust was recognized as a serious disease for wheat production, highly virulent races like Ug99 have been detected on wheat germplasm [2, 3]. These races were observed in Africa and later have been reported from many other parts of the world [4]. Pgt is a macrocyclic‐heterocyclic fungus that completes its life cycle on wheat as the main host and on various species of barberry such as *B. vulgaris*, *B. hispanica*, and *B. garciae* as alternate hosts [5–7]. This fungus produces five different types of spores, including basidiospores, pycniospores (spermatia), aeciospores, urediniospores (uredospores), and teliospores. Pycniospores and aeciospores are produced on barberry leaves and after fertilization of the receptive hyphae with spermatia, dikaryotic hyphae and aeciospores are produced [8]. The presence of a sexual stage on barberry increases the genetic diversity of fungus leading to emergence of new races [9, 10]. Urediniospores migrate long distances through wind and, under favorable conditions, cause epidemics on susceptible wheat cultivars [11].

This disease is mainly controlled using triazole fungicides like cyproconazole, which is a common name of 2‐(4‐chlorophenyl)‐3‐cyclopropyl‐1‐(1H‐1,2,4‐triazol‐1‐yl) butan‐2‐ol compound (Figure S1). Cyproconazole inhibits ergosterol biosynthesis, a crucial component of the fungal cell membrane [12, 13]. Generally, azole fungicides inhibit lanosterol 14‐*α*‐demethylase, an enzyme responsible for converting lanosterol to ergosterol of cell membrane in fungi [14]. Ergosterol is produced from lanosterol through a series of enzymatic reactions involving 51CYP, which is the target of cyproconazole. By binding to 51CYP, cyproconazole hampers the conversion of lanosterol to 4,4‐dimethylcholesta‐8,14,24‐trienol, a precursor of ergosterol, resulting in the accumulation of toxic sterol intermediates and disturbing the membrane permeability and ultimately leading to cell death [15, 16].

Chitosan (CS) is a copolymer derived from D‐glucosamine and N‐acetyl‐D‐glucosamine in a linear form (Supporting Information: Figure S1) obtained through partial deacetylation of chitin. The polymer can be classified as chitin or CS based on the degree of deacetylation determined by the ratio of D‐glucosamine to N‐acetyl‐D‐glucosamine [17]. Various studies show that CS derivatives and CS nanoparticles (CNPs) exhibit antifungal and antibacterial activities and enhance plant resistance against pathogens [18–21]. In numerous studies, CS‐conjugation increased the efficiency, effectiveness, and gradual release of antimicrobial compounds [22–25]. The effectiveness of CNPs and xhitosan‐cyproconazole nanocomposite (Chi‐Cyp) as eco‐friendly alternatives to traditional fungicide cyproconazole against wheat stem was evaluated against the wheat stem rust disease. For this reason, we synthesized and examined the application of CNPs and Chi‐Cyp nanocomposite on disease severity, fungal biomass, and seedling growth.

## 2. Materials and Methods

### 2.1. Biological and Chemical Materials

Wheat seeds of Bolani were obtained from Seed and Plant Improvement Institute in Karaj, Iran. CS (Low Molecular Weight Sigma–Aldrich, deacetylation degree ≥ 75%), sodium tripolyphosphate (TPP) (Sigma–Aldrich), acetic acid 99% (Sigma–Aldrich), cyproconazole (C_15_H_18_ClN_3_O) (purity 95%) from Jiangsu Seven Continent Green Chemical CO., dimethylformamide (DMF) from CARLO ERBA Reagents, Tween 80 (Sigma–Aldrich), RNase (Sigma–Aldrich), and 3,6‐pyridazinediol from Merck, Germany, were used in this experiment. Data analysis was performed using ANOVA and LSD test (*p* ≤ 0.05) using the SPSS 25 software.

### 2.2. Synthesis of CNPs

CNPs were synthesized using the ionic gelation method [26] (Supporting Information: Figure S2A). Half a gram of CS was dissolved in 0.5% acetic acid (100 mL) overnight on a magnetic stirrer (1000 rpm) to obtain a homogeneous solution (0.5%). To improve dispersion and prevent particle aggregation, 1 mL of Tween 80 was added to total 100 mL solution followed by dropwise addition of 0.5% solution of TPP (40 mL) while stirring at 700 rpm [27]. The resulting solution was centrifuged at 30,000 rpm for 30 min, and the obtained pellet was freeze‐dried and stored at 4°C in a desiccator for future use.

### 2.3. Synthesis of Chi‐Cyp

The Chi‐Cyp also was produced using the ionic gelation method [27] (Supporting Information: Figure S2B). Homogeneous solution of CS (0.5%) was prepared as stated above. Cyproconazole in DMF (1%) was solved and added to CS solution. To improve dispersion and prevent particle aggregation, 1 mL of Tween 80 was used. Amount of 40 mL of TPP solution (0.5%) was added dropwise to the Chi‐Cyp solution while stirring (700 rpm). The final solution was centrifuged at 30,000 rpm for 30 min, and the obtained pellet was washed and redispersed in sterile distilled water and freeze‐dried and stored at 4°C [28]. Weight of initial content and pellet in three replicates was measured for analysis. The following formula was used to determine the amount of pellet remaining after centrifugation:

CNPs Reaction Yield RY=Dry pellet weightTotal used CS+TPP weight×100

Chi-Cyp RY=Dry pellet weightTotal used CS+TPP+Cyp weight×100

### 2.4. Dynamic Light Scattering (DLS) Measurements of Nanoparticles (NPs)

Samples were diluted with sterile distilled water, and then DLS was used to measure the hydrodynamic size, polydispersity index (PDI), and zeta potential of NPs using a Zetasizer model, PSS0012‐22 (Malvern, UK). Analysis was performed at a scattering angle of 90° at room temperature. After examining the average size of the NP solution with DLS, field emission scanning electron microscopy (FE‐SEM, MIRA3TESCAN‐XMU) was used to investigate the morphological characteristics of the NPs. Fourier‐transform infrared (FTIR, PerkinElmer Lambda 25, USA) spectroscopy was used to study the functional groups of nanocomposites.

### 2.5. Treatment and Inoculation of Wheat Seedlings

The susceptible wheat cultivar Bolani (Seed and Plant Improvement Institute, Karaj, Iran) was planted in 5 cm plastic pots containing peat moss (10 wheat seeds per pot). Six‐day old seedlings were used for treatment with CNPs, Chi‐Cyp, and other control treatments (spray treatment with 5 mm per pot). Inoculation of fungus was performed with urediniospores of the virulent isolate Pgt99‐65. For inoculation, seedlings were sprayed with water containing 1% Tween 80 and then inoculated with urediniospores using sterile spatulas. Inoculated seedlings were incubated for 18 h in dark at 18 ± 1°C and under 100% humidity. Seedlings were transferred to new condition at 23 ± 2°C with light regime 16 h light and 8 h dark. In this study, seedlings were treated with compounds at 24 h pre‐ and 48‐ and 96‐h post‐inoculation with fungus. All plants were kept in the greenhouse for 14 days at 25 ± 2°C with a relative humidity of 50–60% [29, 30]. At 14 dpi, infection types were scored and classified to low (IF 0 to+2) and high (3–4) indicating the effect of treatments on fungal development [31]. At 14 dpi, leaves containing rust pustules were collected for evaluation of fungal biomass in plant tissue.

### 2.6. DNA Extraction

To assess the fungal biomass, total DNA from all samples using the sodium dodecyl sulfate (SDS) method [32] was extracted. Seedlings of susceptible cultivar Bolani containing stem rust pustules were harvested and were grinded in Chinese mortars using liquid nitrogen. Amount of 1 mL extraction buffer was added to 200 mg of powdered tissue in 2 mL microtubes, and homogenized mixture was incubated in water bath at 65°C for 1 h. Every 10 min, tubes were gently inverted. Then, tubes were centrifuged at 14,000 rpm for 15 min. Supernatant was transferred to a new 2 mL tube, and an equal volume of phenol:chloroform:isoamyl alcohol (25:24:1 [vol/vol]) was added. After gentle homogenization, tubes were centrifuged at 14,000 rpm for 10 min. Supernatant was transferred into new 2 mL tube and homogenized with equal volume of chloroform: isoamyl alcohol (24:1). After centrifugation at 10,000 rpm for 10 min, supernatant was treated with 3 µL of RNase (10 mg/mL) at 45°C for 30 min. Then, sodium acetate (10% of volume) was added to each sample followed by addition of equal volume of isopropanol to microtube. Samples were centrifuged for 10 min at 10,000 rpm, and pellet was washed sequentially twice with 100% and 70% ethanol and dried for 30 min. Amount of 50 µL of deionized water was added to pellet, and DNA concentration was measured by Nanodrop (Nabi Ultraviolet‐Visible Nano Spectrophotometer, Micro Digital Co., Ltd.).

### 2.7. Quantification of Fungal Biomass in Inoculated Plants Using qPCR

To evaluate the fungal biomass of infected plants, we used quantitative polymerase chain reaction (qPCR) (Applied Biosystems StepOne Real‐Time PCR). The standard curve was initially created with ct values obtained from qPCR on dilution series of pure DNA of fungus (0.05, 0.5, 5, and 50 ng). To quantitively determine the amount of fungal biomass in the treated leaves, ITS primer pair of Pgt was used in qPCR using DNA extracted from collected samples. The ct values obtained from qPCR show the percentage of fungal DNA in 100 ng of total DNA [33]. For this research, ITS‐1F (5′‐ TCCGTAGGTGAACCTGCGG ‐3′) and ITS‐4R (5′‐ TCCTCCGCTTATTGATATGC‐3′) were used [34]. The qPCR analysis was performed using the cycling condition: initial denaturation for 10 min at 95°C, 40 cycles of 15 s at 95°C, 30 s at 60°C, and 40 s at 72°C [35]. Data were collected at the end of extension.

### 2.8. Investigating the Effect of NPs on Plant Growth

We evaluated the impact of CNPs and Chi‐Cyp on the morphology and plant growth of treated seedlings. 3 days after germination, germinated seedlings were treated with suspension of 50 μg/mL of NPs. Treatments were performed using three methods: irrigation, spraying, and combined method of irrigation with spraying. Seedlings were grown in 5‐cm pots containing peat moss and incubated at 25°C. The NPs were dissolved in deionized water. For irrigation, 10 mL of NP solution was added to each pot, and for spraying, 5 mL was sprayed on the 3‐day old seedlings in each pot. Distilled water was used for the treatment of control plants. Sampling of treated and control plants was conducted 2 weeks after germination [36–38].

## 3. Results

### 3.1. Reaction Performance, Morphology, and Size Confirm Molecules as NPs

Evaluation of reactive yield (RY) of formulated nanomolecules revealed that 85% ± 5% of CNPs and 87% ± 2% of Chi‐Cyp contributed to the synthesis of CNPs and Chi‐Cyp, respectively. DLS and FE‐SEM methods were used to measure the average size and shapes of NPs. Data showed that the average size of Chi‐Cyp particles (90.11 nm) was slightly larger than that of CNPs, (83.11 nm) as described in Figure 1. The CNPs and Chi‐Cyp particles synthesized in this study were mostly spherical or semi‐spherical, as captured by an electron microscope (Figure 1A,B) CNPs and Chi‐Cyp particles, respectively).

Figure 1(A) FE‐SEM images of synthesized nanomolecules. FE‐SEM shows CNPs are agglomerated together and have formed clusters. (The average size of CNPs was 83.11 nm, ranging from 64.11 to 125.43 nm.) (B) CNPs in combination with cyproconazole (Chi‐Cyp); FE‐SEM shows Chi‐Cyp particles have elliptical to semi‐spherical shape. (The average size of Chi‐Cyp particles was 90.11 nm, ranging from 56.41 to 144.22 nm.) (C) Size distribution by intensity related to CNPs, (D) zeta potential related to CNPs, (E) size distribution of Chi‐Cyp, and (F) zeta potential related to Chi‐Cyp. Size distribution of particles was also measured by DLS in the dissolved state, where solvent molecules (deionized water) interact with the particles. The average size of CNPs (3C) and Chi‐Cyp (3E) was 104.8 and 156.2 nm, respectively. The zeta potential for CNPs was 0.328, while for Chi‐Cyp particles it was 0.238.

**Figure:**
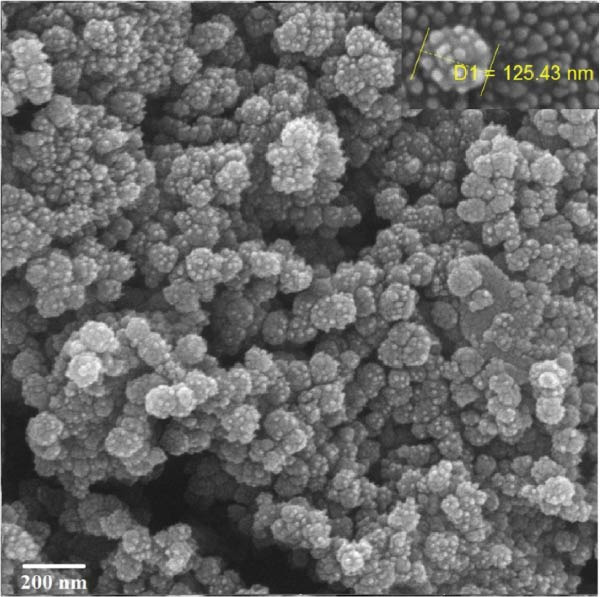
(A)

**Figure:**
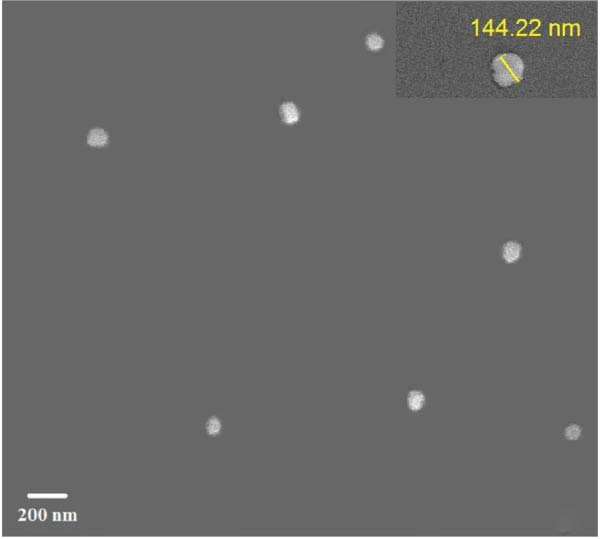
(B)

**Figure:**
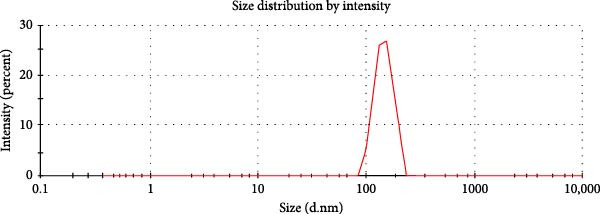
(C)

**Figure:**
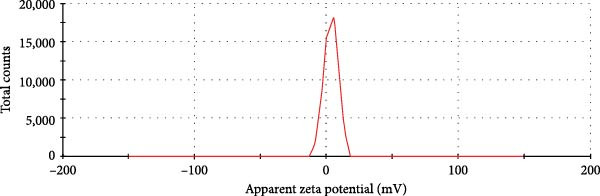
(D)

**Figure:**
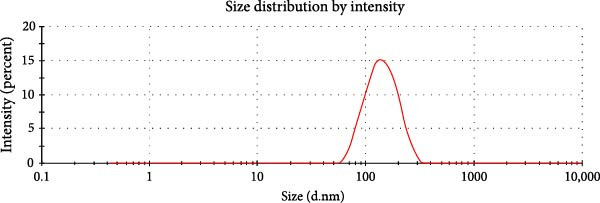
(E)

**Figure:**
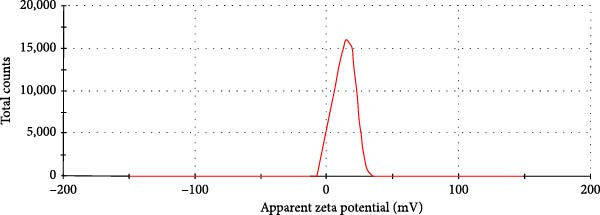
(F)

The PDI and zeta potential were measured alongside other morphological characteristics of the NPs. The PDI and zeta potential are crucial parameters for size uniformity and surface charge of NPs like CNPs, which are essential for various applications in nanotechnology [39]. The PDI and zeta potential for CNPs were 0.328 (Figure 1D) and 14.3 ± 2 mV, respectively, while, for Chi‐Cyp, they were 0.238 (Figure 1F) and 11.3 ± 1 mV.

### 3.2. FTIR Spectroscopy

**Figure 2:**
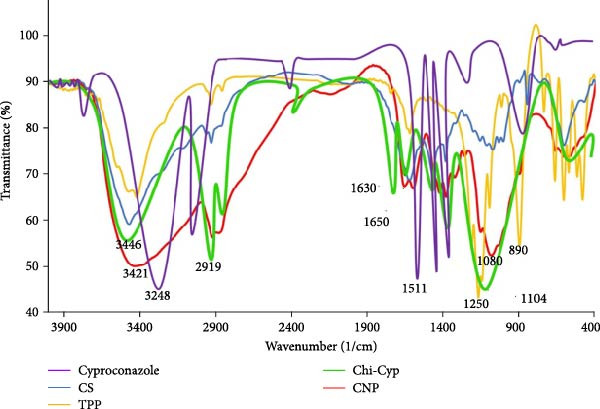
FTIR spectra for various compounds utilized in nanoparticle synthesis. The color‐coded spectra correspond to the FTIR analysis bands for CS, cyproconazole, and the Chi‐Cyp nanocomposite, along with CNPs. Notable absorption bands at ~1380, 890, and 650/cm in the CNPs and Chi‐Cyp spectra are indicative of the C–N, C═C, and C─Cl bonds, respectively. These findings, as reported by Maluin et al. [40], suggest the successful integration of cyproconazole within the CNP framework.

Data represented in Figure 2 show a broad absorption around 3421/cm that belongs to the alcoholic ─OH and ─NH2 groups in the structure of CS. This absorption is enhanced in CNPs indicating strengthened hydrogen bonding. Absorption at 3248/cm belongs to the −OH group in cyproconazole. At 1630/cm, there are sharp and medium absorption in TPP and CS, respectively, which shifts to 1650/cm in the FTIR spectrum of CNPs due to interactions between NH_2_ groups of CS and phosphate groups of TPP. Another clear absorption at 1380/cm belongs to CNPs due to ‐CH_2_ wagging. Absorption at 1250, 1080, and 1104/cm belongs to phosphate groups in TPP, CNPs, and Chi‐Cyp, respectively. Absorption at 1492 and 1511/cm indicates aromatic rings and 3248/cm represents the ─OH group in the Cyproconazole structure [41]. Therefore, absorption at 3446/cm is due to the combination of hydrogen bonding of CNPs and cyproconazole. Absorptions at 2919 and 1104/cm correspond to C–H stretching and C─O stretching vibrations, respectively, while the one at 1383/cm observed in CS, CNPs, and Chi‐Cyp is related to O–H bond [42, 43] (Figure 2). Absorptions at 1380, 890, and 650/cm in CNPs and Chi‐Cyp can be attributed to C–N, C═C, and C═Cl bonds, respectively [40], indicating the attachment of cyproconazole to the CNP matrix.

### 3.3. Application of Nanoparticles Restricts the Stem Rust Disease

Four to 5 days post‐inoculation, chlorotic spots related to colonization of fungus were visible in control plants. To assess their impact, the infection type of disease (IT 1–4) on treated seedlings with cyproconazole, Chi‐Cyp and CNP molecules were evaluated at 2 weeks post‐inoculation (Figure 3A). As shown in Figure 3B, the Chi‐Cyp nano‐formulation had the most significant impact on the development of wheat stem rust symptoms. Treatment at 24 h before inoculation with Chi‐Cyp was not fully successful as inoculation after treatment may remove the treated molecules leading to the development of fungus. Treatment at 48‐ and 96‐h post‐inoculation with 1 μg/mL of Chi‐Cyp successfully restricted the stem rust development (almost no visible symptoms). Treatment of cyproconazole and CNPs was effective at 10 and 100 μg/mL, respectively. There was a significant difference between the treated and control groups (Figure 3B). Comparing the amount of cyproconazole required for full inhibition of disease shows that nanocomposite of fungicide is effective at 1 μg/mL, while fungicide alone is equally effective at 10 μg/mL meaning that 10 times less fungicide is required to control the disease.

Figure 3Representative of disease infection types (IT) of nanomolecules‐treated wheat seedlings. (A) Infection types of stem rust were recorded at 2 weeks post‐inoculation. (B) The average of infection types related to each treatment is indicated in the bar chart. Error bars indicate the variation of data among three biological replications. Infection types were recorded at 24, 48, and 96 hpi (hours post‐inoculation). D and W indicate treatment with solvents and salts used in the experiment (DMF, acetic acid, TPP, NaOH, and water) and sterile distilled water as controls, respectively.

**Figure:**
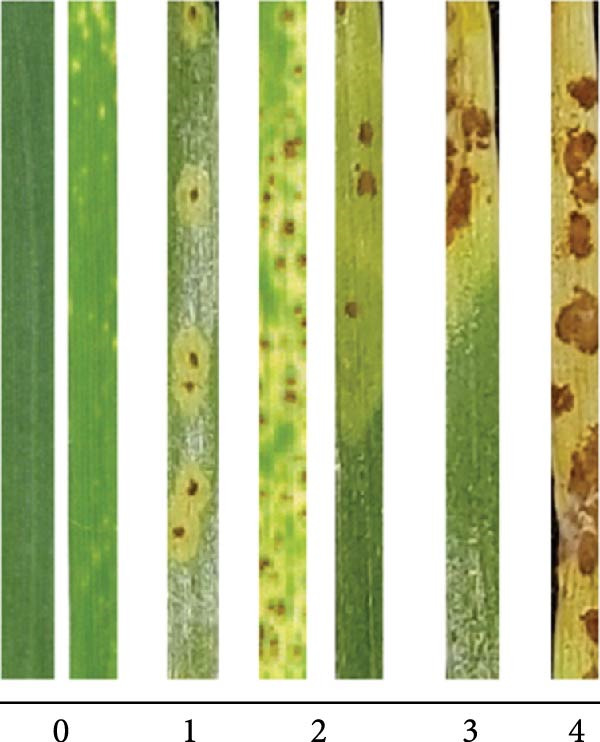
(A)

**Figure:**
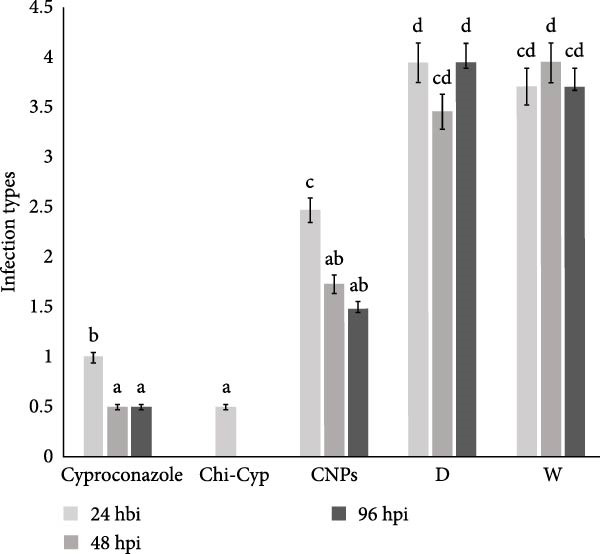
(B)

### 3.4. Application of Nanoparticles Improves the Plant Growth

**Figure 4:**
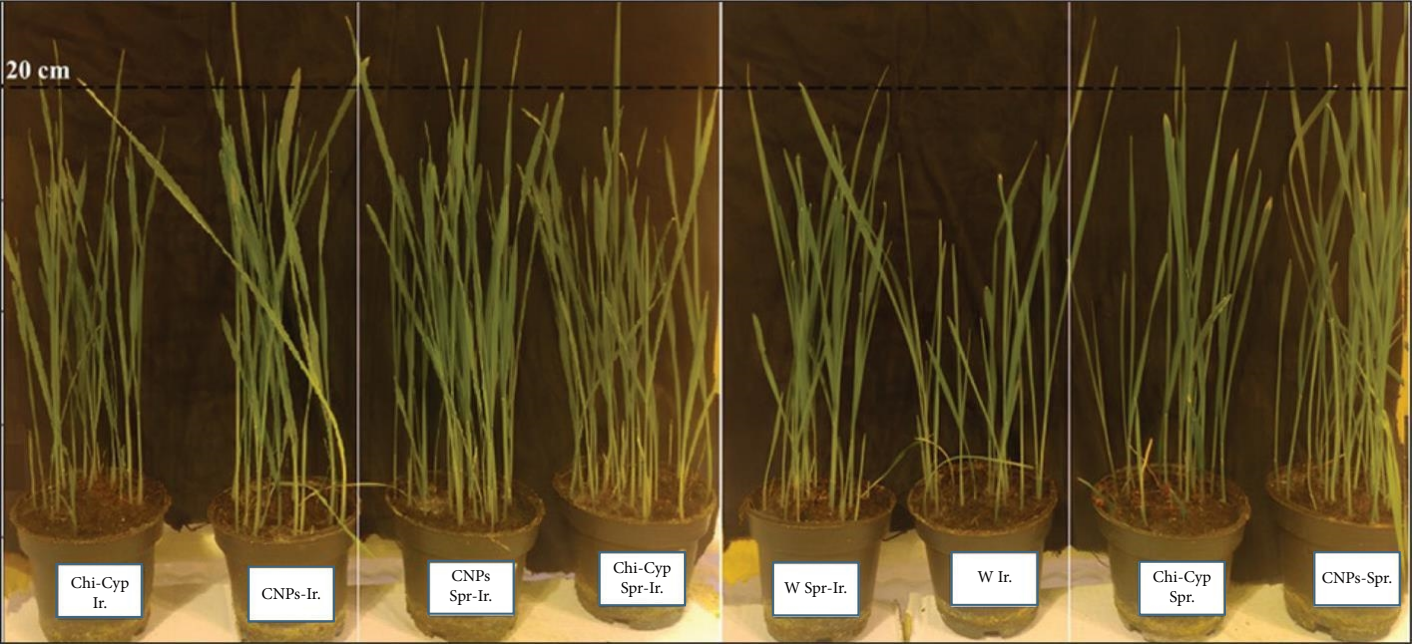
Representation of nanoparticle treatment on plant growth: The plant development when exposed to nanoparticles through distinct application methods: irrigation (Ir.), foliar spraying (Spr.), and integration of both methods (Spr‐Ir.). The use of nanoparticles through irrigation and spraying simultaneously led to further growth in plants. Application of CNP via Spr‐Ir. enhanced the plant growth compared to the Chi‐Cyp nanocomposite and pure water treatments.

**Figure 5:**
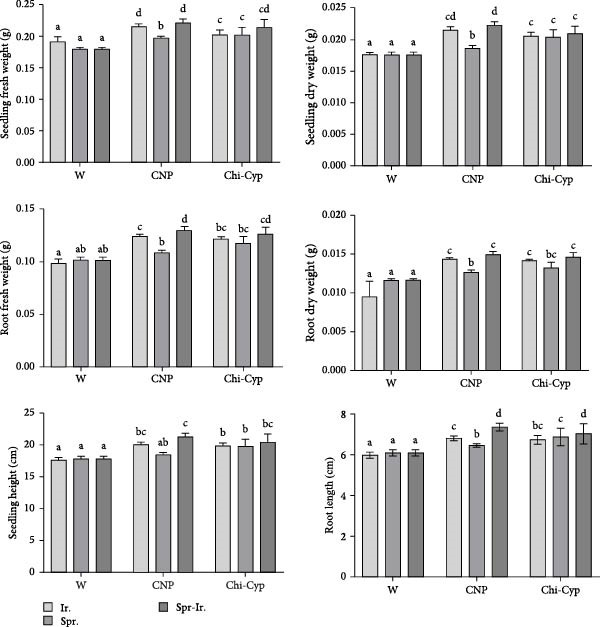
Effect of nanoparticle treatments on growth indexes of plant. Dry and fresh weight of shoots and roots (in g), root length (RL), and seedling Hheight (SH) (in cm) of treated seedlings were measured 2 weeks after nanomolecules treatments. Seedlings were treated with nanoparticles through various application methods: irrigation (Ir.), foliar spraying (Spr.), and integration of both methods (Spr‐Ir.).

To study the effect of NPs on plant growth, seedlings were treated with water (W) and CNPs, either with spraying (Spr.), irrigation (Ir.), or a combination of both methods (Spr‐Ir.). After 14 days post‐inoculation, the fresh and dry weight of shoots and roots as well as the length of shoot and root was measured. Analysis of data revealed a significant difference among treatments. The average of shoot fresh weight (SFW) of water‐treated (W) seedlings through I, S, and SI methods showed a significant difference from those treated with nanomolecules (CNPs and Chi‐Cyp) (Figures 4 and 5). Moreover, the shoot and root fresh and dry weight in the CNP‐treated plants through SI differed significantly from other treatments. In terms of shoot and root length, also the CNP‐treated samples through SI exhibited significant differences compared to all other treatments. Research findings have shown that CNPs and Chi‐Cyp NPs, when applied at 50 μg/mL with a significance level of 5%, have a notable impact on enhancing the average dry and fresh weight of roots and seedlings, as well as promoting the growth of roots and seedlings in the Bolani wheat variety. These results align with prior studies, confirming the positive effects of these NPs on plant development.

### 3.5. Quantification of Fungal Biomass Using RT‐PCR Confirms the Phenotyping Analysis

**Figure 6:**
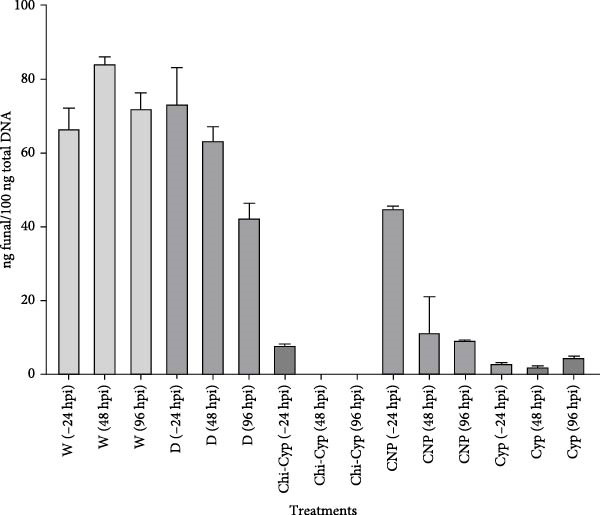
The qPCR measurement of fungal biomass in treated seedlings. The ratio of fungal DNA to the total DNA obtained from wheat leaves infected with the fungus was measured through qPCR analysis. Seedlings were treated at 24 h before inoculation (24 hbi) and 48 and 96 h post‐inoculations (48 and 96 hpi). Samples were harvested 14 days post‐inoculation for DNA extraction. Using qPCR, the standard curve was created based on the dilution series of pure fungal DNA, and the fungal biomass in 100 ng of total DNA was calculated.

To quantify the fungal biomass in treated plants, DNA was extracted from all treatments. Using a dilution series of pure fungal DNA and their related ct values, the standard curve was created. The qPCR data showed that Chi‐Cyp at 1 μg/mL, cyproconazole at 10 μg/mL, and CNPs at 100 μg/mL were effective in reducing fungal biomass (less than 5%) (Figure 6). Mock‐treated plants were fully diseased and contained the maximum amount of fungal biomass (50%–75%). Plants treated at 48 and 96 hpi with Chi‐Cyp showed no detectable symptoms, indicating the absence of fungal growth.

## 4. Discussion

Wheat stem rust due to the emergence of new races is a continuous challenge for wheat farming [44–48]. Therefore, integrated management of disease using resistant cultivars, fungicides, and effective chemicals is required for sustainable wheat production [49, 50]. Data from this study showed that treatment with CNPs (at 100 μg/mL) increased the latent period and reduced the infection rate of fungus as proven previously [51, 52]. CNPs limit the growth, survival, and sporulation of fungus and induce host defense responses [53, 54]. The efficacy of CNPs in restricting the pathogenicity of plant root and leaf pathogens has frequently been demonstrated [55, 56]. CNPs may interfere with the DNA, RNA, and plasma membrane of the fungal cells [57, 58]. The fungicide cyproconazole at 10 μg/mL effectively reduces the infection rate of fungus compared to control treatments. Data showed that the application of Chi‐Cyp nanocomposite at 1 μg/mL was more effective than the separate treatment of CNPs and cyproconazole at their effective concentrations. Apparently, Chi‐Cyp is more effective due to the additive effect of cyproconazole as an inhibitor of ergosterol biosynthesis and CS as the plasma membrane and nucleic acid interfering molecules of fungal cells [59, 60]. In addition to efficacy, the application of Chi‐Cyp drastically reduces the required dosage of cyproconazole (10 times less), which can be economically and environmentally beneficial.

Furthermore, our investigation revealed that the application of CNPs at a concentration of 50 μg/mL significantly enhances the height of the plant, the length of the roots, and both the fresh and dry mass of plant roots and shoots, particularly through methods such as irrigation and foliar spraying. CNPs may be utilized independently or in conjunction with various formulations of nitrogen, phosphorus, and potassium (NPK) fertilizers to promote the growth of plants [61]. Prior research has indicated that CNPs can facilitate seed germination, augment water absorption, activate water channel proteins, and enhance nutrient uptake in plants [62, 63] and consequently improves the plant growth and yield through improving different studied biochemical processes [64]. The NPs of CS exhibit more antifungal activity compared to CS [65, 66]. Although the precise role of cyproconazole in plant growth remains unclear, the application of CNPs in conjunction with Chi‐Cyp presents a promising alternative to traditional fungicides for the management of wheat stem rust. The significant reduction in fungal biomass alongside the beneficial effects on plant development implies that these treatments may result in increased yields, potentially offsetting their initial financial investment. Additionally, the utilization of Chi‐Cyp reduces the quantity of fungicide typically required in conventional applications. Consequently, comprehensive economic analyses should be undertaken to assess whether the advantages in yield and sustainability surpass the financial consequences of implementing these novel approaches in wheat agriculture.

In summary, CNPs enhance wheat growth and facilitate improvements in seed germination, root development, and seedling elongation. The incorporation of CNPs, which are augmented with vital nutrients such as NPK, as well as fungicides including triazoles, enhances their overall efficacy. Moreover, CNPs possess the capacity to activate defense mechanisms and strengthen plant resilience against oxidative stress. The synergistic application of CNPs with cyproconazole represents a novel approach to combatting the wheat stem rust pathogen. By decreasing the quantity of pesticide utilized, the biological risks associated with pesticide application, an inevitable consequence of contemporary agricultural practices, are mitigated. Further investigation is warranted to explore this methodology and to amalgamate pesticides with safer or less toxic alternatives, which could provoke substantial advancements in agricultural output. It is crucial to optimize the concentration and timing of Chi‐Cyp applications to achieve maximal effectiveness in field settings. Collectively, this study indicates that the utilization of Chi‐Cyp holds promise for the mitigation of wheat stem rust and the promotion of wheat growth.

## 5. Conclusion

This study highlights the potential of CNPs and the Chi‐Cyp as effective and environmentally friendly alternatives to traditional fungicides for managing wheat stem rust disease. As reliance on chemical fungicides raises concerns about human health and environmental impacts, incorporating CNPs and cyproconazole into integrated pest management strategies could provide a sustainable solution to improve the resilience of wheat cultivation. The significant reduction in fungal biomass and the enhancement of wheat seedling growth observed with these treatments suggest their promise in combating this serious agricultural threat. Further research is needed to explore their long‐term efficacy and economic viability in diverse agricultural settings.

## Conflicts of Interest

The authors declare no conflicts of interest.

## Author Contributions

**Mansoor Karimi-Jashni, Maryam Nikkhah, and Seyed Ali Moosawi-Jorf**: conceived the project. **Jafar Fathi-Qarachal, Maryam Nikkhah, and Mansoor Karimi-Jashni**: carried out the experimental work. **Jafar Fathi-Qarachal and Mansoor Karimi-Jashni**: wrote the manuscript. All authors have approved the manuscript.

## Funding

The authors received no specific funding for this work.

## Supporting Information

Additional supporting information can be found online in the Supporting Information section.

## Supporting information


**Supporting Information** Figure S1: Chemical structure of Cyproconazole and Chitosan. Figure S2: (A) Synthesis process of CNPs in an aqueous environment using the ionic gelation method. (B) The proposed model for the connection method of Chi‐Cyp particles.

## Data Availability

The data that support the findings of this study are available from the corresponding author upon reasonable request.
