# Supplementary material for: Fabrication, Characterization, and Antifungal Activity of Chitosan–Cyproconazole Nanocomposite for Simultaneous Wheat Stem Rust Control and Growth Enhancement
Source: IET Nanobiotechnol. 2026 Jan 2;2026:6628425. doi: 10.1049/nbt2/6628425 (PMC12782328; doi:10.1049/nbt2/6628425)
Supplement: Supplementary file 1 — Supporting Information Figure S1: Chemical structure of Cyproconazole and Chitosan. Figure S2: (A) Synthesis process of CNPs in an aqueous environment using the ionic gelation method. (B) The proposed model for the connection method of Chi‐Cyp particles. [file NBT2-2026-6628425-s001.docx]

**Supplementary data**

**Fabrication, characterization****, and antifungal activity of Chitosan-Cyproconazole Nanocomposite for simultaneous Wheat Stem Rust Control and Growth Enhancement**

Jafar Fathi-Qarachal, Seyed Ali Moosawi-Jorf, Maryam Nikkhah, Mansoor Karimi-Jashni


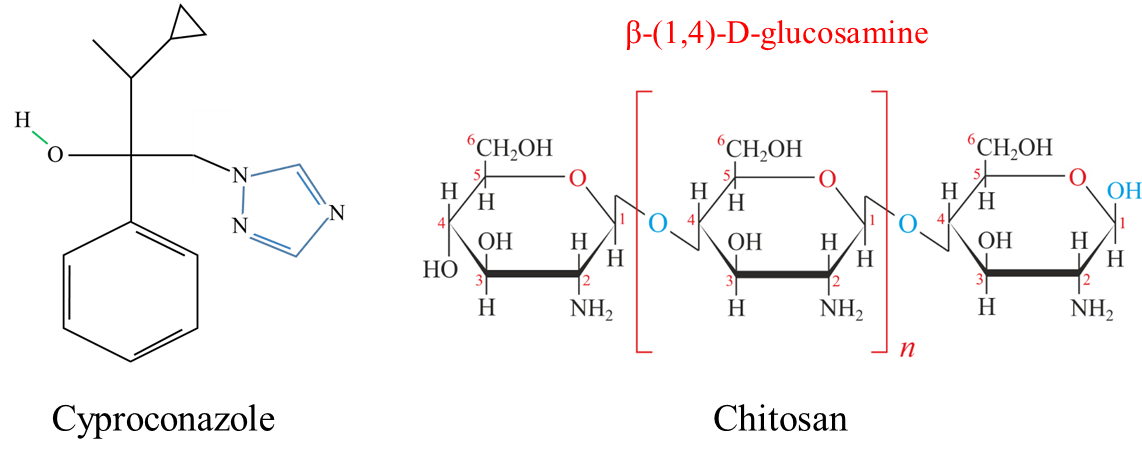


**Figure S1.** Chemical structure of Cyproconazole (Kang et al., 2015) and Cs (Ogawa et al., 2004). The bond depicted as β-(1→4)-glycosidic linkage show links between D-glucosamine units. The “n” in the structure indicates the degree of polymerization (the number of repeating glucosamine units) in the chitosan chain.


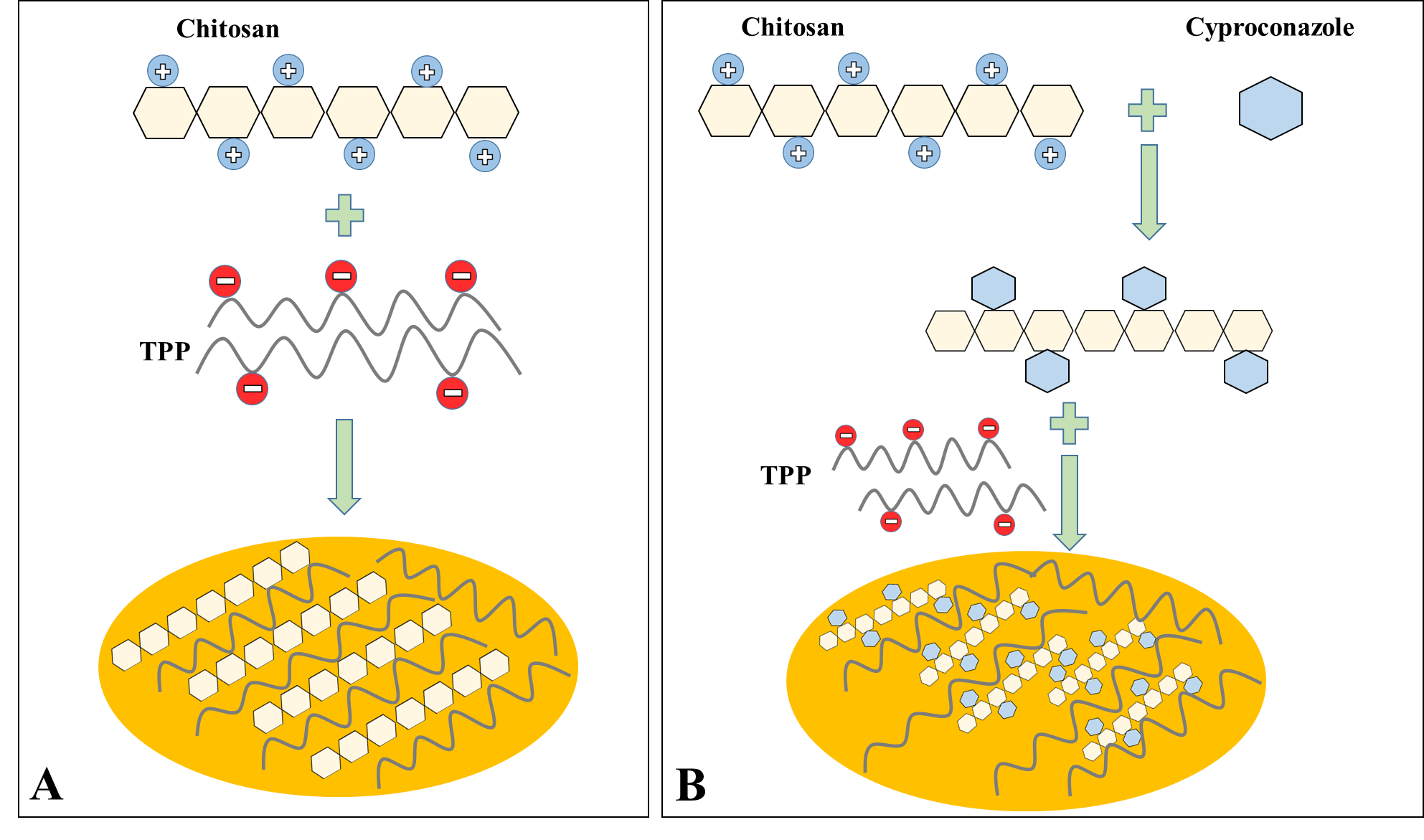


**Figure S2.** **A.** Synthesis process of CNPs in an aqueous environment using the ionic gelation method **B.** The proposed model by Maluin et al. (2019) for the connection method of Chi-Cyp particles.
